# RNA Bricks—a database of RNA 3D motifs and their interactions

**DOI:** 10.1093/nar/gkt1084

**Published:** 2013-11-12

**Authors:** Grzegorz Chojnowski, Tomasz Waleń, Janusz M. Bujnicki

**Affiliations:** ^1^International Institute of Molecular and Cell Biology, Trojdena 4, 02-109 Warsaw, Poland, ^2^Faculty of Mathematics, Informatics, and Mechanics, University of Warsaw, Banacha 2, 02-097 Warsaw, Poland and ^3^Institute of Molecular Biology and Biotechnology, Faculty of Biology, Adam Mickiewicz University, Umultowska 89, 61-614 Poznan, Poland

## Abstract

The RNA Bricks database (http://iimcb.genesilico.pl/rnabricks), stores information about recurrent RNA 3D motifs and their interactions, found in experimentally determined RNA structures and in RNA–protein complexes. In contrast to other similar tools (RNA 3D Motif Atlas, RNA Frabase, Rloom) RNA motifs, i.e. ‘RNA bricks’ are presented in the molecular environment, in which they were determined, including RNA, protein, metal ions, water molecules and ligands. All nucleotide residues in RNA bricks are annotated with structural quality scores that describe real-space correlation coefficients with the electron density data (if available), backbone geometry and possible steric conflicts, which can be used to identify poorly modeled residues. The database is also equipped with an algorithm for 3D motif search and comparison. The algorithm compares spatial positions of backbone atoms of the user-provided query structure and of stored RNA motifs, without relying on sequence or secondary structure information. This enables the identification of local structural similarities among evolutionarily related and unrelated RNA molecules. Besides, the search utility enables searching ‘RNA bricks’ according to sequence similarity, and makes it possible to identify motifs with modified ribonucleotide residues at specific positions.

## INTRODUCTION

Folded RNA molecules exhibit hierarchical organization. They are composed of modular units, in particular regularly shaped double-stranded helices formed by ribonucleotide residues paired in the Watson–Crick (WC) sense, and irregularly shaped motifs formed by residues engaged in various non-WC interactions. Examples of structural motifs include kink-turn (1), sarcin–ricin motif (2), π-turn (3) and t-loop (4,5). These motifs usually have complex internal structures, and they participate in interactions of high biological significance. They often introduce precise kinks and turns of the RNA backbone that position adjacent helices with respect to each other, and they mediate specific intra-molecular contacts that induce the compact folding of medium-sized and large RNAs (6). They also frequently form binding sites on the surface of RNA molecules that are responsible for interactions with proteins, small molecule ligands and with other RNAs (reviews: (7–9)). Consequently, the understanding of RNA structure–function relationships depends critically on the identification and classification of the motifs, both in terms of their internal structure and with respect to the molecules they interact with.

Experimental structure determination for a growing number and type of RNAs revealed that structural motifs are often conserved in homologous (evolutionarily related) molecules, but they may also appear in different structural and functional contexts in non-homologous molecules. Consequently, structural motifs are not necessarily accompanied by a conserved RNA sequence or secondary structure, hence their discovery and comparison is not trivial (3).

Currently, several databases that classify the RNA structural motifs exist. Some of them provide also information about tertiary interactions in RNA molecules. SCOR (10) is a manually curated RNA 3D motifs database that provides both structural and functional classification. It is, however, no longer updated. RNA Frabase 2.0 (11) stores RNA secondary structure elements (stems, loops) and their spatial coordinates. Its search algorithms enable making primary and secondary structure queries, including kissing loop interactions; they also enable making backbone geometry queries. RLoom (12) and RNA CoSSMos (13) databases are large collections of RNA 3D motifs, both providing an interface to the symbolic motif search tool MC-Search (14). RNAJunction categorizes internal loops and junctions formed by up to nine helices and kissing loops (15). Another valuable tool is the DARTS database (16) based on the ARTS program for pairwise RNA structure comparison (17). It focuses on classification of structural similarities between known RNA structures and enables making user-defined queries. Finally, a recently released RNA 3D Motif Atlas provides detailed and partially curated information about sequence variability and secondary structure of RNA 3D motifs (18). This database provides also some insight into the structural environment of motifs. Users may display and download protein and RNA residues that are within 16 Å from a selected RNA motif. These residues are derived from biological assemblies defined in the Protein Data Bank (PDB), but not those that are neighbors due to crystallographic contacts. Furthermore RNA 3D Motif Atlas provides secondary structure diagrams for a representative set of ribosomal RNAs with an interactive mapping to known motifs. It has an accompanying tool WebFR3D (19) that enables searching for RNA structural motifs within structures stored in the database. There are also databases that provide data specifically on intra-molecular contacts involving RNA. A database of metal ions in nucleic acids (MINAS) compiles detailed information on all metal ions found in available structures of nucleic acids (20). MINAS enables detailed searches to be made based on the ion coordination environment. On the other hand, Nucleic acid–Protein Interaction Data Base (NPIDB) (21) stores information about all available structures of DNA–protein and RNA–protein complexes.

Despite great efforts spent on RNA structural motif recognition and classification, there are several issues that remain unsolved. The first problem is that the molecular environment of the RNA structure models available in PDB is rarely taken into account. In particular, information about contacts between symmetry mates in a crystal is often ignored, despite its influence on local features of RNA structure and in some cases even on the global fold of the RNA. Besides, for large structures containing RNA molecules (e.g. the ribosome), models are split into several PDB files that are not necessarily independent. For example the asymmetric unit of one of the crystal structures of the 70S ribosome is composed of four PDB files (id codes: 4KFH, 4KFK, 4KFL, 4KFI) (22), but only two of them contain coordinates of all ions in the whole structure (see Supplementary Example S2 in Supplementary Data 1 for details). Another issue is the common practice of using the maximum resolution of diffraction data as a measure of structure quality. It is well known, however, that crystal structure model quality is a local property and must be locally validated (23).

We developed a database named RNA Bricks to resolve the above-mentioned issues. Our database provides information about local environments of the collected motifs, including contacts with other RNA motifs, proteins, metal ions, water molecules or small molecule ligands. Furthermore, RNA Bricks stores data on contacts between symmetry mates in crystals and between molecules from split PDB entries (i.e. in huge structures divided into multiple files, due to the PDB file format restrictions). A unique feature of RNA Bricks is the availability of three structure-quality scores with single-nucleotide resolution. These may be used to select most reliable subsets of stored RNA structures and motifs. We also implemented an algorithm for PDB-wide structure-based searches. The algorithm has similar capabilities to the above-mentioned WebFR3D, but additionally enables making PDB-wide queries.

## MATERIALS AND METHODS

### Definition of an RNA Brick

**Figure 1.**
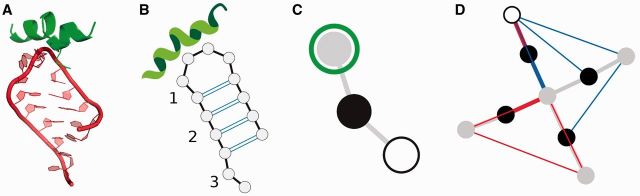
RNA stem-loop structure (**A**) presented in a standard secondary structure representation (**B**) and reduced graph representation used in RNA Bricks (**C**). Nodes of the reduced graph correspond to the RNA 3D motifs: gray—loops (B.1), black—helical stems (B.2), open circle—terminal fragments (B.3). Gray edges denote shared nucleotide residues or pairs, red and blue edges correspond to the intra-molecular and crystallographic contacts, respectively. Green circles indicate motifs that are in contact with protein. tRNA structure (PDB: 1EHZ) in a reduced graph representation (**D**).

The term ‘RNA (3D) motif’ has many meanings (7,24). In this work, we introduce a specific definition of an ‘RNA brick’ as a set of interacting nucleotide residues from the same chain, flanked by WC or wobble base pairs. In particular we distinguish three types of motifs. Stems are arrays of WC/wobble base pair tandems. Loops are motifs composed of single-stranded fragments flanked by WC/wobble pairs. Terminal fragments are single-stranded fragments with only one end involved in a WC/wobble pair (Figure 1B).

### Implementation

Utility programs were implemented in Python 2.7 with the extensive use of routines from the Computational Crystallography Toolbox (CCTBX) (25). The database web interface was developed in the Django framework (http://djangoproject.com). The visualization of reduced RNA graphs is based on a JavaScript InfoVis Toolkit (http://thejit.org). Secondary structure representations of RNA motifs were rendered using an in-house modified version of Varna (26), which enables visualization of stacking interactions and multiple-chain structures. Interactive 3D representations of RNA motifs are displayed in Jmol (http://www.jmol.org/).

### Data preparation

Models of macromolecular structures containing RNA molecules (with or without proteins), excluding DNA/RNA hybrids, were downloaded from the PDB (27). Structures that possess at least two covalently bound unmodified ribonucleotide residues in all-atom representation (with or without hydrogen atoms) were used to populate the RNA Bricks database. Experimental diffraction data for crystallographic structures were downloaded when available and converted to the binary MTZ file format using the cif2mtz program from CCP4 suite (28).

### RNA motifs extraction and clustering

For each RNA chain found in a PDB model, secondary structure was annotated with MC-Annotate (14). After removing pseudo-knots with K2N (29), the base pairing information was converted to the secondary structure graphs with nodes representing ribonucleotide residues and edges representing either phosphodiester bonds (RNA backbone) or WC/wobble base pairs. Next, we applied the minimum cycle basis algorithm (30,31) to detect motifs in the graph, that is helical stems and unpaired fragments (in the sense of the absence of classical secondary structure), either internal or terminal. The algorithm finds a minimum collection of cycles (i.e. closed paths where no node appears twice), which can be used to construct any cycle in the graph. In case of RNA secondary structure graphs (without pseudo-knots), cycles that are tandems of base pairs correspond to helical stems, and the remaining cycles represent various types of loops. It is important to emphasize that in this approach any pair of adjacent motifs shares at least one common ribonucleotide residue. Finally, we extracted atomic coordinates of the motifs, grouped them by type (stem, loop or terminal fragment) and composition (i.e. number, length and order of continuous RNA chain fragments). For each group a hierarchical clustering process was used to identify geometrically similar motifs with <1.0 Å RMSD calculated for backbone atoms following optimal superposition. As a result, we obtained clusters composed of RNA motifs with the same number of ribonucleotide residues.

### Tertiary structure-based search algorithm

The input to the tertiary structure-based search algorithm are a query RNA structure *q* and a set of RNA 3D motifs *M* = {*m*_1_, *m*_2_, *m*_3_, … *m*_n_}. The task is to find a set of rigid transformations that superposes *M* members onto *q* with lowest RMSD over backbone atoms. An additional restriction is that all *m*_i_ nucleotides must have their counterpart in the query structure *q*. A symmetric version of the problem, where we want to find all matches of *q* within *M* members, is handled in the analogous way.

The algorithm starts with a two-step procedure of filtering motifs from *M* that cannot match *q*. First, we compare distances between selected backbone atoms within motifs from *M* and the structure *q*. At this step the carbon C3′ atoms are used by default. The user, however, may request the use of any other backbone atom type. All motifs from *M* that have distances not observed in *q* are rejected. Next, for structure *q* and remaining motifs from *M* we analyse all possible triplets of the selected backbone atoms. Again, we reject all motifs that do not contain at least one triplet with similar edge distances to a triplet from *q*. Finally, for each of the remaining motifs we pick a triplet of the selected backbone atoms, and try to superimpose it onto all similar triplets from the structure *q*. The obtained transformation is applied to the whole motif and quality of the match is scored with the RMSD of the closest pairs of the selected backbone atoms from *q* and *m*_i_ after superposition. If the RMSD is below a user-provided threshold, the superposition is further refined with the use of all backbone atoms.

### Nucleotide contact detection

Putative hydrogen bonds were detected using our own utility scripts developed with the CCTBX library. We used our own implementation of the algorithm from the HB-PLUS program (32). Missing hydrogen atoms were added to the structures with Reduce (33).

The detection of putative interactions of RNA with molecules other than RNA and proteins, i.e. with water, ions and small molecules, was based on a simple distance-based criterion. Two neighbors were classified as being in contact if the smallest distance between their non-hydrogen atoms was below 3.9 Å.

### Base pair annotations

Base pairs were annotated using MC-Annotate (14), RNAView (34), FR3D (35) and our own scripts based on detected H-bonds and definitions from (36). Putative stacking interactions were additionally annotated using code adapted from ModeRNA (37,14). Since the three secondary structure annotation methods listed above use different naming schemes, we used a unified nomenclature based on a consensus approach, as implemented in our own method ClaRNA (T.W., G.Ch, J.M.B., manuscript submitted, http://iimcb.genesilico.pl/clarna/).

### Quality scores

All nucleotide residues in RNA bricks are annotated with structure-quality scores that describe real-space correlation coefficients (RSCCs) with electron density data (if available for crystallographic structures), backbone geometry and possible steric conflicts. These scores can be used to identify poorly modeled residues and to assist in the selection of well-modeled motifs. The measure of steric clashes, i.e. a number of non-H-bond overlaps (0.4 Å or greater) per 1000 atoms, was determined with the use of the Probe program (38) from the Molprobity suite (39). Suspicious backbone torsion angles were detected based on a set of 54 favorable RNA backbone conformers defined by the RNA Ontology Consortium with the use of the Suitename program from the Molprobity suite (39). A fraction of nucleotide residues with poor electron density was derived from experimental structure factors deposited in PDB (if available). Poor electron density should be interpreted either as a weak signal (below 1 σ on average) or as RSCC value below 0.7. RSCC is a standard real-space fit quality measure used in crystallography [e.g. in MAPMAN (40)]. Parameters defining a low-quality fit used in this work were selected arbitrarily, based on our experience in analysing RNA structures. All three scores were calculated both for complete RNA content of a given PDB entry (global score), and for separate RNA motifs (local score).

## RESULTS

### The database web interface

**Figure 2.**
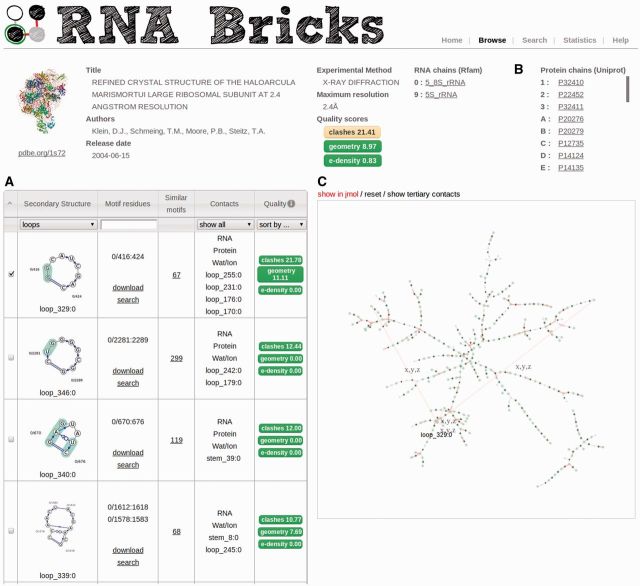
The RNA Bricks web interface displaying details of the *H. marismortui* large ribosomal subunit (PDB: 1S72). Green halos on VARNA diagrams (**A**) depict ribonucleotide residues that are in contact with proteins.

The RNA Bricks web interface provides intuitive access to the data on RNA 3D motifs, i.e. ‘RNA bricks’ (Figure 2). The user may browse a catalog of structures or query the database with a PDB (27), Rfam (41) or Uniprot (42) identifier (Figure 2B). RNA bricks are listed in interactive tables that display sequence and secondary structure data, local quality scores and contact information (Figure 2A). Annotated interactions between pairs of motifs are listed explicitly with links to the secondary structure visualization. Contacts between symmetry mates and molecules from split PDB entries are additionally highlighted. Additionally, selected RNA brick coordinates may be downloaded in the PDB format together with a text file that contains a list of interactions.

#### Visualization of RNA 3D motifs

3D structure of RNA bricks and their complete local environment (including symmetry mates and neighboring molecules from split PDB entries) can be visualized with the Jmol applet. Users may toggle visibility of selected types of neighboring molecules (RNA, protein, ligand and water/ion). Contact with protein and RNA molecules are presented down to the level of single hydrogen bonds (see Materials and Methods section for details).

#### Sequence-based search

The database enables searching for RNA motifs based on sequence and secondary structure similarities. Queries must be in the FASTA format, and define sequences of continuous RNA chain fragments that form a motif. The search engine supports regular expression queries and accounts for cyclic permutations in the order of segments. By default, the sequence-based search is case-insensitive. The users, however, may select *case sensitive* option to search for modified residues that are represented by a lower-case symbol of a related residue (in this work we follow the scheme used in RNAView, e.g. ‘u’ stands for m5U, m2U and any other uridine modification). Additionally the query results that are within 1.0 Å backbone RMSD distance may be aggregated. This option may be useful if a number of results is very large.

#### 3D structure-based search

The RNA Bricks database may be queried with an RNA structure in PDB format comprising up to 40 nt residues. Two search modes are available; *query-in-motif* searches for instances of the query structure within RNA motifs, whereas *motif-in-query* attempts to cover the query structure with motifs from the database. Because of the complexity of the structure-matching algorithm, the exhaustive search involving all the available motifs is computationally prohibitive. Therefore a single query is limited to representatives of the RNA 3D motif clusters (see Materials and Methods section for details). These can be simply medoids (default), motifs derived from a set of non-redundant RNA structures (43), or motifs that form a selected type of contacts (e.g. with proteins, RNA or ligands). Users may also search a set of fragments derived from a selected PDB entry.

### RNA motifs extraction and clustering

The database records are updated weekly with each new release of the PDB and recent statistics are presented on the RNA Bricks webpage. As of 15 August 2013, RNA Bricks stored 2573 structures that contain RNA molecules (97% of structures with RNA chains available in PDB), and 220 460 RNA 3D motifs. A majority of these are loops (51.8%). In particular terminal loops constitute 15.9%, internal loops 28.2%, three-way-junctions (3wj) 4.2% and loops composed of more than 3 strands 3.5% of all the RNA 3D motifs. Canonical helices (double stranded) and single-stranded terminal fragments comprise 40.6% and 7.6% of all RNA 3D motifs, respectively. After applying the clustering procedure (see Materials and Methods section for details), we obtained 16 089 motifs that represent clusters of RNA 3D motifs with the RMSD of backbone atoms ≤ 1.0 Å. Fraction of representative stem motifs is 13.4%, which is significantly smaller than overall fraction of stems in the database. All types of loops constitute 63.2% of representatives. Majority of these are internal loops (32.4% of all representatives). Internal loops, 3wj and loops composed of more than three strands constitute 20.5%, 5.7% and 4.6% of all representatives, respectively. Relatively large fraction of the representatives (23.4%) are single-stranded terminal fragments.

### Quality scores

To assess the mutual dependence of the quality scores used in RNA Bricks we calculated the Spearman's rank correlation coefficient for all pairs of score values. For the calculations we used only RNA motifs derived from crystallographic structures that have experimental data deposited in the PDB.

The correlation coefficients for all three pairs of scores calculated for complete RNA structures are 0.42 for poor electron density and clash-score, 0.38 for poor electron density and suite-score and 0.44 for clash-score and suite-score, respectively. These correspond to relatively low, but statistically significant correlations. Analogous correlation coefficients determined for RNA motifs are 0.35, 0.28 and 0.31, which again corresponds to weak, but statistically significant correlation. These results suggest that the three quality scores should be used together, as they contribute complementary information, but they should not be treated as completely independent from each other.

### Example: tertiary interactions involving the T-loop motif

**Figure 3.**
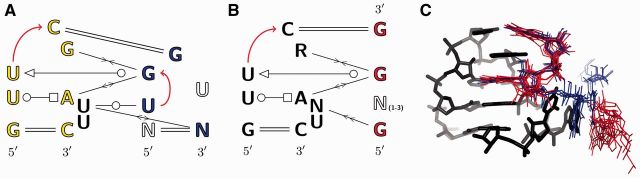
Two interaction modes involving T-loop in tRNASec (**A**) and tRNA (**B**) Rfam families. Yellow letters depict query nucleotides, *R* denotes a purine and *N* any nucleotide. The right-side figure (**C**) presents superposition of all the high-quality fragments from the non-redundant set of RNA-containing crystallographic structures solved at 3.0 Å resolution or better. Only one representative of the T-loops is depicted (black lines). Blue and red lines represent conserved nucleotides observed in tRNASec and tRNA families, respectively.

The RNA Bricks was developed to enable studies of RNA tertiary interactions. To demonstrate its capabilities we analyzed contacts formed by the conserved T-loop motif. The T-loop (Figure 3) is a recurrent RNA motif, known to be involved in a variety of tertiary interactions in many RNA families (4,5). A large cavity formed between the fifth and sixth nucleotide residue in this motif is capable of accepting an intercalating base that can additionally interact with the exposed sugar edge of the third nucleotide residue. This complex interactions network enables formation of stable contacts that connect sequentially distant parts of RNA molecules or enable formation of stable intermolecular interactions.

The most conserved part of the T-loop from a high-resolution X-ray structure (PDB: 1EHZ, residues A/53,54,55,56,57,58,61, yellow letters in Figure 3A) was used to search the RNA Bricks database with default parameters, *query-in-motif* mode and RNA motifs derived from a non-redundant set of X-ray structures solved at 3.0 Å resolution or better. The results were manually curated with the use of RNA Bricks web interface to remove hits that matched the query with low RMSD, but were not T-loops (e.g. had fifth and sixth nucleotide stacked with each other). Families of structures not covered by the current release of Rfam database were assigned using Infernal (44) and the Rfam (41) covariance models.

In total we found matches within 10 RNA 3D motif clusters, which correspond to 76 unique RNA motifs from 9 Rfam families. These are tRNA (47), tRNA-Sec (7), SSU_rRNA_eukarya (5), TPP (5), SSU_rRNA_bacteria (4), FMN (2), 5_8S_rRNA (3), AdoCbl-variant (2) and group-II-D1D4-3 (1). A complete list of results is available at http://iimcb.genesilico.pl/rnabricks/algorithms/browse_result/tloop_search_curated/. Due to limitations of space in this article we will discuss in detail only contacts involving highest quality motifs (i.e. with all quality scores below 25.0) from the first match. Among 20 motifs, 16 were derived from RNA structures annotated as tRNA, 3 as tRNASec and 1 as group-II-D1D4-3 (PDB: 4FAW). In both tRNA and tRNASec families, the third uridine residue and the apex cytosine residue of the motif interact with conserved guanine residues (Figure 3A and B). The two families, however, have different binding modes involving flipped-out nucleotide residues at positions 7 and 8. In the tRNA family motifs, a residue at the seventh position is stacked with a conserved guanine. In contrast, in tRNASec motifs, the seventh residue is additionally paired with uridine. An exception is a T-loop motif from tRNA(Asp) (PDB: 1IL2), which forms interactions similar to the tRNASec family. We also found a T-loop like motif in a representative of a group-II-D1D4-3 family (PDB: 4FAW). In spite of the structural similarity to the T-loops in tRNA, this motif lacks a very characteristic WC pair involving the apex nucleotide (45). This observation is consistent with the proposed structural basis of the tRNA recognition (46).

## DISCUSSION

In this work we described RNA Bricks, a database of recurrent RNA 3D motifs and their interactions. Unlike other similar tools, RNA Bricks provides detailed information about the local environment of RNA motifs, including contacts with other RNA motifs, proteins, metal ions, water molecules and ligands. Furthermore, data available uniquely in RNA Bricks are contacts between symmetry mates in crystals and molecules from split PDB entries (i.e. divided into multiple files, due to the PDB format restrictions). RNA Bricks provides also three structure-quality scores with a single-nucleotide resolution. These may be used to select the most reliable subsets of stored RNA structures and motifs. We also implemented an algorithm for making PDB-wide structure-based queries. In contrast to other similar tools RNA Bricks accepts user-provided queries in PDB format. Besides, the search utility enables searches for ‘RNA bricks’ according to sequence similarity, and makes it possible to identify motifs with modified ribonucleotide residues at specific positions.

To address the problem of secondary structure visualization of large RNA molecules (e.g. ribosomes) we developed the reduced graph representation. The graphs make it possible to display complex interaction networks (e.g. between ribosomal subunit and tRNA) and provide efficient mapping between secondary structure elements and the RNA 3D motifs (Figure 1).

### How to use the RNA Bricks database

RNA Bricks database provides a simple clustering method that groups RNA motifs with the same secondary structure and number of ribonucleotide residues. Therefore in some cases two motifs that have similar tertiary structures are classified to separate clusters due to differences in secondary structure annotation. Furthermore, very similar 3D motifs with variable loops (e.g. T-loop described above) do not belong to the same clusters. Being aware of these limitations we implemented a structure search algorithm that allows for comparison of RNA structure fragments according to the mutual position of individual atoms and regardless of the secondary structure and the molecule size. Users should utilize this tool to query the database for all RNA 3D motifs that share common substructures with a motif of interest.

### Tertiary structure-based search algorithm

We compared the structure-based search tool implemented in RNA Bricks to other publicly available web servers that enable PDB-wide searches using coordinates of small, user-defined RNA fragments. To the best of our knowledge among currently available methods only R3D-Blast (47), WebRF3D (19) and FASTR3D (48) fulfill these criteria. WebFR3D and FASTR3D accept as queries only fragments of PDB structures that are stored in a database accompanying these tools. R3D-Blast allows searches to be made for structures uploaded by a user. We intentionally excluded from the comparison the backbone search methods such as RNA Frabase 2.0 that require sugar pucker amplitude and torsion angle ranges to be provided as an input, because these are relatively difficult to define for custom fragments.

Our solution of the 3D motif search problem is similar to the algorithm implemented in ARTS (17) for the comparison of large RNA molecules. The major difference, however, is that we do not use any secondary structure information for computing initial superposition. Therefore, the input structures may be incomplete, or be represented in a coarse-grained fashion. Thus, RNA Bricks can be used to identify tertiary motifs in structures generated by RNA 3D structure modeling methods that do not use full-atom representation, such as NAST (49), DMD (50) or SimRNA (51). This clear advantage of the algorithm comes with a tradeoff of an increased computational complexity, and makes our approach computationally costly for the comparison of large RNA structures. However, RNA fragments stored in RNA Bricks are relatively small (average size of a fragment is 11 nt) which results in a reasonable performance. The actual computation time depends on both the query size and server load. In our tests, searches with relatively large RNA fragments composed of 20 nt residues took 15 min on average.

In this work we showed that the RNA Bricks motif search tool reproduces results reported previously for FR3D (35) (see Supplementary Example S1 in Supplementary Data 1 for details). All occurrences of a sarcin–ricin motif loop, including non-local ones, were found within *Haloarcula marismortui* 50S ribosomal subunit (PDB: 1S72). In addition, we were able to carry out the search for all known RNA structures, which was not possible using the web version of FR3D (35), WebFR3D (19). We also attempted to perform an analogous search with the FASTR3D (48) and R3D-Blast (47) web interfaces. Both these methods, however, found no matches.

### Contacts involving the T-loop motif

We used the RNA Bricks structure-based search tool and web interface to elucidate interaction patterns that involve a conserved T-loop motif. First, we listed all occurrences of this motif in RNA families that have at least one representative with known, high-resolution 3D structure. Subsequently, we described in detail tertiary interactions that involve the T-loop motif in tRNA families. Most of these interactions were already described in the articles that reported individual structures; however, the search with the use of the RNA Bricks database interface allowed for a comprehensive comparative analysis to be made.

### Quality scores

RNA Bricks stores three structure-quality scores with a single-nucleotide resolution, which can be used to select most reliable subsets of RNA structures and motifs. These scores are: backbone geometry reliability (suitescore), the presence of severe steric clashes (clashscore) and low RSCCs for experimental diffraction data (if available, for crystallographic structures only). In this work we showed that although the three scores are not independent, the correlation between them is relatively weak. Notably, RNA motifs that correspond to well-resolved electron density maps do not necessarily have favorable RNA backbone conformers (data not shown). Therefore we suggest that users should always take all the available quality scores into consideration. RNA Bricks provides the RSCCs determined for all structures with experimental data deposited in the PDB, including those, for which refinement parameters reported by the authors cannot be reproduced. It means that in some cases the overall low correlation coefficient may reflect gross errors in the diffraction data deposited by their authors, rather than the actual quality of a structure. In borderline cases users should refer to specialized tools like the Electron Density Server (52) or PDB_REDO (53).

## SUPPLEMENTARY DATA

Supplementary Data are available at NAR Online.

## FUNDING

Polish Ministry of Science [Iuventus Plus 0066/IP1/2011/71 to G.Ch.]; European Research Council (ERC) [StG grant RNA+P=123D to J.M.B.]; the development of the substructure search tool by T.W. has been supported by the National Science Centre (NCN) [2011/01/D/NZ1/00212 to G.Ch.]. Funding for open access charge: Polish Ministry of Science [Iuventus Plus 0066/IP1/2011/71].

*Conflict of interest statement*. None declared.
